# Integrating autonomic and affective pathways in borderline personality disorder: the triangle therapy hypothesis

**DOI:** 10.3389/fpsyg.2026.1686068

**Published:** 2026-02-02

**Authors:** Daniel Juraszek

**Affiliations:** Independent Researcher, Milton Keynes, United Kingdom

**Keywords:** affective neuroscience, autonomic nervous system regulation, borderline personality disorder, developmental trauma, exposure therapy, neuroaffective therapy, polyvagal theory

## Abstract

**Introduction:**

Borderline personality disorder is marked by emotional lability, unstable identity, and hypersensitivity to abandonment. Although mainstream treatments, such as dialectical behavior therapy, schema therapy, and mentalization-based therapy, reduce symptoms, they often bypass the subcortical affective systems shaped by early attachment trauma. This conceptual paper presents triangle therapy for borderline personality disorder, a neuroaffective intervention based on the premise that three ancestral affective conditions—silence, sound, and isolation—are hypothesized to shape autonomic dysregulation in borderline personality disorder.

**Method:**

Triangle therapy for borderline personality disorder proposes a 30-session protocol involving progressive exposure to each condition over ten sessions. Stimulus duration would increase from 5 to 50 min under continuous therapist attunement without verbal interpretation. The model emphasizes embodied co-regulation to support potential autonomic integration of historically overwhelming affective states.

**Hypothetical results:**

Potential outcomes may include recalibration of vagal and sympathetic tone, extinction of catastrophic prediction errors, and emergence of symbolic-affective processing. The model integrates polyvagal theory, affective neuroscience, and psychodynamic frameworks on early neglect. Safety protocols and inclusion criteria are specified to support empirical evaluation.

**Discussion:**

Triangle therapy for borderline personality disorder is a theoretical model proposed to inform future research and clinical development. It is not a replacement for existing evidence-based treatments but is framed as a somatic pre-phase that may improve affective tolerance and therapy engagement. Empirical testing through pilot studies and multimodal physiological assessment is essential before clinical implementation. The protocol aims to provide new opportunities for treating severe affective instability in outpatient and inpatient settings.

## 1 Introduction

Borderline personality disorder (BPD) presents significant challenges in both clinical treatment and research contexts. Individuals who meet criteria for BPD often cycle through emergency departments, outpatient programs, and unstable relationships, burdened by affective lability, self-injurious behaviors, and chronic fears of abandonment. Recent epidemiological reviews report that borderline personality disorder occurs in approximately 0.7–2.7% of the general adult population—with markedly higher rates observed in clinical settings—reaching roughly 10–20% among psychiatric outpatients and inpatients in high-income Western regions, including Europe and North America (Leichsenring et al., 2024; Widiger and Trull, 2007). Even clinicians with specialized training often report feeling overwhelmed by the rapid emotional shifts and intense relational dynamics characteristic of BPD presentations.

Over the past three decades, several evidence-based interventions have been developed, notably dialectical behavior therapy (Linehan, 1993), schema therapy (Young et al., 2003), and mentalization-based treatment (Bateman and Fonagy, 2010). These models have demonstrated efficacy in reducing self-harm, enhancing emotion regulation, strengthening attachment security, and fostering cognitive restructuring. However, approximately 25–30 percent of patients discontinue treatment before completion (Barnicot et al., 2011), and even among those who remain in therapy, enduring remission is relatively uncommon, with chronic emptiness and affective instability frequently persisting over time (Zanarini et al., 2010).

Recent approaches also demonstrate attempts to regulate subcortical affect directly. Compassion-focused therapy has shown promise in reducing threat-based arousal through parasympathetic activation and self-soothing imagery (Gilbert, 2014), while HRV-biofeedback interventions aim to increase vagal tone and autonomic stability, providing an emerging physiological pathway relevant to BPD (Lehrer et al., 2020; Krause Utz et al., 2019). From a right-brain developmental perspective, early implicit affect regulation systems provide the neurobiological backdrop for later psychotherapeutic work, including models that aim to engage autonomic and subcortical processes more directly (Schore, 2012).

From a developmental neurobiological perspective, the infant nervous system is shaped not by conceptual reasoning, but by patterned sensory-affective exchanges with caregivers. Predictable warmth, prosodic vocalization, and attuned silence convey safety to the autonomic system, supporting the maturation of affiliative and autonomic networks (Feldman, 2017; Carozza and Leong, 2021). In contrast, inconsistent cues or emotional unavailability condition threat responses. Meta-analytic data indicate that individuals later diagnosed with BPD are substantially more likely to report emotional neglect and abuse in early caregiving environments, consistent with chronic exposure to such inconsistent or aversive cues (Porter et al., 2020). Affective neuroscience research highlights that subcortical structures such as the amygdala, hypothalamic–pituitary–adrenal axis, and periaqueductal gray consolidate these patterns long before cortical regions can narrate them (Damasio, 1999; Porges, 2011). Experimental paradigms of social exclusion show that individuals with borderline personality disorder exhibit heightened rejection sensitivity and altered pain processing when excluded (Bungert et al., 2015), helping to explain why, for some, a non-response to a text message may evoke a threat-level autonomic reaction and quiet relational moments may feel more like abandonment than safety.

Clinical reports and qualitative interviews similarly emphasize that patients often describe not isolated traumatic events but persistent emotional atmospheres: the suffocating silence of the family kitchen, a caregiver's unpredictable yelling, or prolonged solitude after perceived misbehavior (Miller et al., 2021; Baptista et al., 2021). These affective memories often emerge as somatic distress—accelerated heart rate, visceral anxiety, or derealization—well before language is accessible. Traditional talk therapies may name these sensations but seldom resolve them at their origin. There is a risk, therefore, of intellectualizing what is essentially an embodied state of survival threat lodged in the autonomic nervous system.

This conceptual paper introduces triangle therapy for borderline personality disorder (TTB), a theoretical model. It proposes that three core affective conditions—silence, sound, and isolation—function as foundational sensory-emotional climates that shape borderline dysregulation. Drawing on dimensional models that distinguish deprivation (absence of expected input) from threat (intrusive, unpredictable input), silence corresponds to emotional absence, sound to chaotic auditory stimulation, and isolation to persistent loss of social contact (McLaughlin et al., 2014; Teicher and Samson, 2016). Together, these three conditions capture lack of contingent response, intrusive acoustic threat, as well as relational disconnection. They are repeatedly linked to childhood maltreatment, alterations in fronto-limbic and sensory networks, with elevated risk for borderline and related psychopathology (Porter et al., 2020; Tomoda et al., 2011; van Harmelen et al., 2010).

Other sensory modalities, such as touch or olfaction, may also be clinically relevant, but TTB treats this triad as a parsimonious, testable starting point rather than an exhaustive taxonomy of early adversity. In this framework, silence may have been experienced as non-existence, sound as chaos, and isolation as abandonment. Over time, the nervous system may learn to respond to these states with panic, dissociation, or protest behaviors. TTB hypothesizes that therapeutic change may involve structured, titrated re-engagement with these conditions within a regulated clinical context, allowing them to be re-encoded as tolerable and survivable.

TTB synthesizes findings from polyvagal theory, affective neuroscience, and attachment-trauma literature. Polyvagal theory emphasizes that safety and danger are detected pre-consciously through the autonomic nervous system, particularly the ventral and dorsal branches of the vagus nerve (Porges, 2011). Attachment-focused psychophysiological studies further report systematic heart rate variability changes during attachment activation and link attachment insecurity to reduced vagal flexibility and heightened defensive arousal (Farina et al., 2015; Zingaretti et al., 2020; Pourmand et al., 2023), suggesting that insecure attachment and early neglect may bias neuroception toward defensive postures, limiting access to social engagement systems. Complementary research demonstrates that affective experiences consolidate in the limbic system prior to cognitive narration (Damasio, 1999; LeDoux, 2002), while trauma literature suggests that overwhelming early states fragment sensory and memory processing (van der Kolk, 2014). TTB integrates these findings into a structured progression: exposure → containment → re-patterning.

In practice, TTB can be conceptualized as a 30-session protocol divided into three modules—Silence, Sound, and Isolation—each hypothetically consisting of ten sessions. In this illustrative framework, exposure durations might increase from 5 to 50 min across sessions. Crucially, these are not exposures to brief, discrete stimuli but to immersive affective environments, with Silence, Sound, and Isolation functioning as labels for broader affective climates rather than single cues. Silence is established by eliminating verbal interaction and ambient sound; sound exposure uses calibrated tones (e.g., specific frequencies in Hertz with increasing volume); isolation involves brief periods in which the therapist leaves the room but remains observable through one-way glass or intercom. Between exposures, the therapist facilitates grounding techniques such as breath regulation, proprioceptive tapping, or gentle movement—without cognitive processing. In the hypothetical protocol, verbal meaning-making would be deferred until the completion of each module to prioritize embodied integration.

The rationale for this somatic-first approach is supported by extinction learning research, which indicates that safe, repeated exposure is necessary to recalibrate prediction errors encoded in limbic circuits (LeDoux, 2002). Overexposure or lack of containment may reinforce these circuits, increasing rather than reducing reactivity. Thus, TTB carefully titrates exposure intensity, mirroring dose–response principles found in prolonged exposure therapies. However, it has two key distinctions: (1) the target is internal physiological states rather than external cues; and (2) cognitive narration is postponed to avoid disrupting somatic learning.

The therapist's role within TTB diverges from directive interventions such as DBT. Instead, the clinician maintains a regulated, non-intrusive presence—a “co-regulatory anchor”—signaling safety through physiology rather than instruction. In line with accounts conceptualizing BPD as marked by epistemic mistrust and pervasive difficulties in trusting interpersonal communication, this anchoring stance is not assumed to depend on pre-existing trust but is offered consistently across sessions as a scaffold for its gradual restoration (Fonagy et al., 2015, 2017; Preti et al., 2023).

Process research further indicates that, even in brief treatments for BPD, therapeutic alliance and trust tend to evolve across the first sessions and are sensitive to how therapists respond to patients' relational needs, rather than representing fixed prerequisites for treatment (Kivity et al., 2020). In TTB, early distrust, testing, and alliance ruptures are therefore anticipated, and the therapist's task is to maintain a predictable, non-retaliatory physiological and relational posture across exposure modules. This presence fulfills a fundamental biological expectation for co-regulation in early development. Between modules, biofeedback (e.g., heart rate variability [HRV]) may be used to track autonomic progress and reinforce the client's awareness of internal shifts.

Importantly, TTB is not proposed as a replacement for established treatments, nor is it a standalone intervention. Rather, it is envisioned as a preparatory phase that enhances the nervous system's capacity for later relational, cognitive, or skills-based work. Some patients may enter DBT with increased emotional bandwidth; others may experience symptom reduction sufficient to pursue goals outside of psychotherapy. Determining TTB's placement within a stepped-care model remains an empirical question.

The remainder of this article proceeds as follows: the next subsection reviews neurodevelopmental mechanisms underlying early affective imprinting; Sections 3 and 4 outline the TTB tools and protocol in operational detail; Section 5 examines hypothesized mechanisms of change; and Section 6 discusses clinical applications, limitations and directions for research. The author's intent is not to assert a definitive intervention but to propose a model for empirical testing and critical dialogue. If future evidence supports its efficacy, TTB may offer a novel contribution to the early-phase treatment of severe affective dysregulation. All procedural elements and parameters described in the following sections should therefore be understood as provisional, hypothetical scaffolds for future empirical studies, not as validated clinical prescriptions.

## 2 Theoretical background

Borderline personality pathology is widely conceptualized as a disturbance in the integration of affect regulation, identity coherence, and attachment organization (Fonagy et al., 2018; Schore, 2003). In a meta-analysis of 47 cohort studies, Porter et al. (2020) demonstrate that early relational adversity—including emotional neglect, inconsistent caregiving, and disorganized parental responses—predicts the emergence and severity of borderline traits more reliably than any specific genetic polymorphism identified to date. Furthermore, the authors reported an odds ratio of 6.2 for childhood neglect and 9.4 for emotional abuse in individuals later diagnosed with BPD, compared to non-clinical controls. These findings support a developmental neurobiological view, emphasizing the role of early embodied adaptations in the etiology of borderline dysregulation. TTB builds on this perspective, positing that three elemental affective states—silence, sound, and isolation—become encoded in subcortical circuitry as foundational survival expectations.

### 2.1 Early sensory-affective imprinting

The mammalian neonate is born with an incomplete nervous system that requires co-regulatory interactions to reach functional maturity. Auditory, tactile, and visual cues—such as maternal heartbeat, rocking, vocal prosody, and gaze synchrony—entrain the infant's neural oscillations, fostering myelination of the ventral vagal pathways and establishing a physiological baseline for safety (Feldman, 2017). In contrast, prolonged silence—for example, a caregiver who is emotionally withdrawn—deprives the infant of contingent feedback necessary for affective attunement. Similarly, exposure to unpredictable, high-intensity sounds or long intervals of social disconnection calibrates the developing nervous system toward hypervigilance or collapse. These early affective patterns form a somatic template for future threat detection and regulatory strategies.

Animal studies corroborate this mechanism: rat pups raised by low-licking/grooming dams exhibit exaggerated hypothalamic-pituitary-adrenal reactivity in adulthood (Francis et al., 1999), while rhesus monkeys subjected to repeated maternal separations display patterns consistent with dorsal vagal dominance, as evidenced by bradycardia and behavioral hypoactivity (Suomi, 1997). In primates, these grooming and close-contact behaviors constitute an early tactile affiliative channel that is conserved in humans through affective touch mediated by C-tactile afferents. Affective touch studies indicate that interpersonal trauma and borderline personality pathology are associated with altered evaluation of pleasant caress-like touch, increased touch aversion, and atypical integration of painful and pleasant tactile stimuli (Strauss et al., 2019; Spitoni et al., 2020; Löffler et al., 2022; Cruciani et al., 2023, 2025).

Translational imaging studies in humans show parallel patterns at the neural level: adults with histories of emotional neglect or disorganized attachment demonstrate increased amygdala reactivity to emotional or neutral stimuli, greater amygdala volume, and atypical resting-state connectivity between the amygdala, hippocampus, anterior cingulate cortex, and related limbic structures (van Harmelen et al., 2010; Lyons-Ruth et al., 2016; van Hoof et al., 2019; Cruciani et al., 2021). These neurobiological correlates are not incidental—they trace the developmental trajectory by which early tactile-affiliative experiences, and their absence, are embedded in autonomic patterns.

### 2.2 Polyvagal theory and neuroception of threat

Polyvagal theory (Porges, 2011) delineates two branches of the vagus nerve: a phylogenetically older unmyelinated dorsal branch associated with immobilization and a newer myelinated ventral branch implicated in social engagement. Polyvagal accounts of vagal regulation have been criticized for overly deterministic interpretations of vagal pathways, limited alignment with comparative neuroanatomy, and conceptual conflation of respiratory sinus arrhythmia with a simple index of cardiac vagal tone (Grossman and Taylor, 2007; Liem and Neuhuber, 2021). The model is therefore used here as a heuristic framework rather than as a settled description of autonomic circuitry. “Neuroception” refers to a rapid, non-conscious process by which the nervous system evaluates safety or danger through cues such as facial expression, tone of voice, and acoustic environment. Within the TTB framework, the following neuroceptive pairings are tentatively hypothesized rather than empirically established one-to-one correspondences:

1. **Silence** → neuroception of abandonment → dorsal vagal collapse
2. **Chaotic sound** → neuroception of intrusion → sympathetic arousal
3. **Prolonged isolation** → neuroception of irrevocable aloneness → oscillation between sympathetic protest and dorsal shutdown

These associations are understood as developmentally shaped patterns that may be repeatedly reinforced during early caregiving and are therefore proposed as testable hypotheses rather than established neurophysiological facts. Consequently, seemingly innocuous adult experiences—such as being ignored in a conversation, hearing a loud noise, or spending time alone—may reactivate autonomic states formed in infancy, often without conscious awareness.

### 2.3 Right hemisphere dominance and implicit memory

Developmental neuroscience indicates that the right hemisphere—which mediates prosody, interoception, and affect appraisal—matures before the left hemisphere, which governs language and sequential reasoning (Schore, 2012). Early trauma is therefore encoded predominantly in right-hemispheric somatosensory representations rather than left-hemispheric verbal narratives. Ogden et al. (2006b) describe this as the tyranny of procedural memory, in which the body continues to act as though threat is imminent even when the cognitive mind recognizes the current context as safe.

### 2.4 Triadic architecture of dysregulation

TTB conceptualizes borderline dysregulation as emerging from a three-part schema rooted in affective experience, proposed here as a conceptual model rather than an empirically established typology:

- **Silence**

° *Cue*: absence of auditory or emotional resonance
° *Autonomic reflex*: may involve bradycardia, shallow respiration, derealization
° *Implicit belief* : “I disappear; I cease to exist.”

- **Sound**

° *Cue*: unpredictable, high-frequency auditory stimuli (e.g., >600 Hz)
° *Autonomic reflex*: may involve tachycardia, hypertonia, cortisol surge
° *Implicit belief* : “Chaos is imminent; I must brace or erupt.”

- **Isolation**

° *Cue*: prolonged absence of mutual gaze, proximity, or touch
° *Autonomic reflex*: may involve initial sympathetic protest followed by dorsal shutdown
° *Implicit belief* : “No one will come; existence is solitary and unsafe.”

These nodes are conceptualized as patterns that frequently co-occur and reinforce one another. For instance, silence may precede a caregiver's sudden yelling, followed by imposed isolation. Over time, this triad may form a closed-loop system that, in this framework, is hypothesized to consolidate into the core symptom cluster of BPD: affective instability, abandonment sensitivity, identity disturbance, defined here as a chronic disruption of a continuous sense of self, and impulsivity.

### 2.5 Limitations of cognitively centric therapies

While evidence-based interventions such as DBT, schema therapy, and mentalization-based treatment provide valuable cognitive and interpersonal tools, their efficacy depends on the client's ability to remain within an optimal arousal window. Neuroimaging studies suggest that individuals with BPD may exit this window more rapidly than healthy controls and, in some paradigms, other clinical groups. For example, fMRI work has reported prolonged amygdala responding during instructed fear processing, and diminished habituation or prolonged return-to-baseline patterns during repeated exposure to emotional stimuli in BPD relative to healthy controls (Kamphausen et al., 2013; Hazlett et al., 2012; Koenigsberg et al., 2014). Without autonomic access, cognitive strategies may fail to integrate, particularly under emotional duress. Moreover, emerging work on non-verbal and physiological synchrony between therapists and clients suggests that moment-to-moment alignment of movement and autonomic signals has been associated with stronger therapeutic alliance and better outcomes. Such synchrony is treated as a conceptual, testable hypothesis that will require direct measurement in future empirical studies (Koole and Tschacher, 2016; Ramseyer and Tschacher, 2011; Tschacher and Meier, 2020).

### 2.6 Neuroscience of graded exposure

Fear extinction research shows that learning is optimized when a stimulus fails to confirm an expected threat, thereby inducing “prediction error” and modifying synaptic connections in the basolateral amygdala and ventromedial prefrontal cortex (Milad and Quirk, 2012). The following parameters are proposed as testable hypotheses rather than empirically established prescriptions. TTB operationalizes this principle through three exposure axes:

1. **Frequency Axis (Sound Module)** Tones begin in mid-range (e.g., 400 Hz at 40 dB) and progress toward 700 Hz at 65 dB to simulate, yet regulate, early auditory trauma.
2. **Amplitude Axis (Silence and Isolation Modules)** “Amplitude” is indexed by exposure duration, ranging from 5 to 50 min, and is intended to enable disconfirmation of the belief that silence or solitude equals annihilation.
3. **Relational Axis** Throughout exposure, the therapist maintains steady, non-intrusive presence—modulating breath, posture, and gaze—to anchor neuroception in safety.

### 2.7 Co-regulation as external vagal support

Human infants regulate arousal via co-regulation with caregivers; they cannot self-soothe independently. In adulthood, many individuals with BPD lack this internalized regulatory function. Within TTB, the therapist operates as an external ventral vagal signal—a live cue of safety. Biofeedback studies suggest that physiological synchrony between therapist and client, such as heart rate coherence, predicts reductions in dissociative symptoms over subsequent hours (Feldman, 2020). TTB is thus conceptualized as offering relational exposure, not simply sensory desensitization.

### 2.8 Symbolic integration following somatic mastery

According to the somatic marker hypothesis (Damasio, 1999), bodily states could be mapped by interoceptive centers such as the insula and anterior cingulate before cognitive narratives can emerge. The hypothesis remains influential yet contested, with critical reviews questioning the specificity of peripheral feedback signals in complex decision making and cautioning against oversimplified one-to-one mappings between somatic states and cognitive processes (Dunn et al., 2006; Linquist and Bartol, 2013). TTB respects this sequence by deferring verbal interpretation until the conclusion of each ten-session module. At this stage, patients may spontaneously produce metaphoric language, for example describing quiet as becoming a blanket instead of a coffin, which can indicate integration of somatic experience into autobiographical memory.

### 2.9 Complementarity with existing trauma models

TTB aligns with bottom-up approaches such as somatic experiencing, sensorimotor psychotherapy, and structural dissociation theory (Payne et al., 2015; Ogden et al., 2006a). Its unique contributions include (1) defining a fixed triad—silence, sound, isolation—as the minimum affective scaffold for developmental trauma; (2) specifying dosing parameters (e.g., frequency, amplitude, duration) suitable for clinical manualization and research reproducibility; and (3) mandating co-regulatory presence (“anchor witness”) as a core procedural component so that exposures are intended to be consistently buffered by social safety. In the isolation module, self-regulation is practiced in physical solitude but within an internalized relational frame established by the therapist's prior and subsequent co-regulatory presence (Porges, 2022).

### 2.10 Working hypotheses for empirical testing

The following hypotheses remain to be tested empirically. Meta-analytic evidence suggests reduced resting vagal tone in BPD, indexed by vagally mediated heart rate variability, consistent with autonomic inflexibility in low-stimulation contexts (Koenig et al., 2016). Resting vagally mediated HRV could be indexed primarily using the root mean square of successive differences (RMSSD).

Experimental studies link HRV and dissociation to physiological reactivity during stress tasks, supporting vagal indices and sympathetic markers, such as galvanic skin response (GSR), as candidate endpoints (Krause Utz et al., 2019). Ecological momentary assessment (EMA) research indicates that affective instability, stressor-linked shifts in affect, and self-harm urges can be captured in daily life, providing ecologically valid metrics for generalization (Trull et al., 2008; Chaudhury et al., 2017). Adult Attachment Interview outcomes, including narrative coherence and reflective functioning, can change over the course of intensive treatment and therefore constitute plausible narrative endpoints (Levy et al., 2006). Symptom measures such as the BSL-23 are sensitive to therapeutic change and capture emptiness and related dimensions (Bohus et al., 2009).

Here, “AAI coherence” denotes coherence and reflective functioning scores derived from the Adult Attachment Interview, “BSL-23” denotes the 23-item Borderline Symptom List, and “Zanarini score” refers to the total score on the Zanarini Rating Scale for Borderline Personality Disorder (ZAN-BPD; Zanarini et al., 2003). Recent trials of compassion-based and physiology-informed adjunct interventions in BPD, as well as work integrating wearable physiology into personality disorder treatment, suggest that modules targeting emptiness and autonomic regulation are clinically relevant and measurable in trial settings (Casellas Pujol et al., 2024; Krause Utz et al., 2019; Lans et al., 2025). These studies do not test TTB, but they justify the outcome domains specified below. Accordingly, the following clusters are offered as working hypotheses requiring direct testing and potential falsification prior to any clinical application.

**Cluster 1 – autonomic specificity**

- *H1a (Silence)*: individuals with BPD may show greater dorsal vagal dominance (reduced RSA, bradycardia) and higher micro-dissociation scores during silent laboratory conditions than control groups.
- *H1b (Sound)*: when exposed to graduated tones (400–700 Hz, 40–65 dB), BPD participants may exhibit stronger sympathetic activation (e.g., galvanic skin response [GSR], startle reflex) with slower recovery.
- *H1c (Isolation)*: monitored isolation may elicit a biphasic response—initial sympathetic activation followed by vagal collapse—more prominently in BPD than in clinical controls.

**Cluster 2 – dose-dependent adaptation across 30 sessions**

- *2a (Silence)*: progressive extension of silence may increase vagal tone and reduce dissociative events by session 10.
- *2b (Sound)*: incremental auditory dosing may lower peak GSR and cortisol half-life.
- *2c (Isolation)*: gradual exposure may delay protest onset and reduce post-exposure vagal suppression.

**Cluster 3 – generalization to daily life**

- *3a*: post-Silence module, patients may report reduced evening emptiness via EMA.
- *3b*: post-sound module, EMA may show fewer noise-triggered self-harm urges.
- *3c*: post-isolation module, passive sensing may reveal increased time spent alone without affective collapse.

**Cluster 4 – narrative emergence**

- *4a*: AAI coherence scores may improve for narratives involving quiet or solitude.
- *4b:* storylines involving sound trauma may show improved thematic cohesion.
- *4c*: accounts of isolation may shift from fragmented to integrated autobiographical narratives.

**Cluster 5 – add-on efficacy within DBT**

- *5a*: DBT preceded by the Silence module may show faster reduction in the BSL-23 emptiness subscale.
- *5b*: DBT with Sound module priming may accelerate reductions in anger rumination.
- *5c*: DBT following the Isolation module may improve distress tolerance, with Zanarini scores ≥ 0.6 SD below controls by week 16.

## 3 Implementation requirements

This section defines falsifiable, measurable variables to support future empirical testing of TTB. The technical parameters below function as research-grade placeholders to enable disconfirmation as well as confirmation. They do not imply feasibility, tolerability, or clinical safety, and they are not intended as a treatment manual or practice guideline. Total therapeutic dose could consist of 30 individual sessions, each lasting approximately 60 min. These could be delivered once or twice weekly over ~12–30 weeks, consistent with intensive psychotherapies for BPD and conceptual accounts linking therapeutic change to repeated cycles of memory reconsolidation (Alberini and LeDoux, 2013; Lane et al., 2015). This schedule is proposed as a pragmatic scaffold rather than an empirically established reconsolidation interval. These sessions could be divided into the following three sequential modules:

- **Module I** – **silence**

° *Core affective theme*: annihilation anxiety, existential void.
° *Therapeutic goal*: establish a tolerable and embodied sense of existence in the absence of external stimuli.

- **Module II** – **sound**

° *Core affective theme*: intrusive chaos, fear–rage admixture.
° *Therapeutic goal*: foster sympathetic regulation under calibrated auditory stimulation.

- **Module III** – **isolation**

° *Core affective theme*: abandonment panic, dissociative drift.
° *Therapeutic goal*: maintain coherence and self-containment during physical solitude.

As one possible research proposal, exposure durations could be arranged to progress in a modestly non-linear fashion across sessions (e.g., 5, 7, 10, 15, 20, 25, 30, 35, 40, and 50 min). This sequence was selected heuristically to implement a titration-like progression from brief to longer exposures, consistent with inhibitory learning accounts emphasizing prediction error and expectancy violation (Craske et al., 2008, 2014). All parameters are illustrative and subject to empirical testing and safety evaluation.

### 3.1 Infrastructure and tools

Facility requirements could include a soundproof or acoustically treated room with ambient noise ≤ 30 dB; calibrated clinical-grade headphones or flat-response monitors (20 Hz-20 kHz); a decibel meter to verify exposure accuracy; a one-way glass panel; and an adjacent monitoring station with live video feed. There could be a primary therapist, a backup clinician for emergencies, and a technical assistant to manage the biosignal equipment. GDPR/HIPAA-compliant encryption should also be possible for biometric data storage and retrieval. Protocols should be in place for seizure, psychotic decompensation, suicidality, or vasovagal collapse. Furthermore, informed consent should explicitly address non-standard exposure procedures; patients retain the right to pause or discontinue at any point.

The following tools would be required in a future clinical evaluation:

- **HRV:** RMSSD via chest strap; illustrative upward trend ≥10 ms.
- **GSR**: peak frequency and amplitude tracked across sessions.
- **Salivary cortisol**: collected via passive drool into polypropylene tubes at T1 (−10 min), T2 (+0 min), T3 (+30 min); samples stored frozen (e.g., at −20 °C) until batch assay; illustrative downward trend in area under the curve.
- **Machine learning prediction**: mixed-effects modeling forecasts dropout risk; red flag triggered by HRV decline diverging from subjective distress for >3 sessions.
- A real-time dashboard for HRV (RMSSD), GSR peaks, and respiratory rhythm.

### 3.2 Therapist stance and core competencies

The therapist should have the following competencies:

- **Anchored presence**

° Breathing rate approximately 4–6 bpm.
° Relaxed postural alignment (external shoulder rotation, soft jaw, open gaze directed at the upper sternum).
Peripheral visual awareness to detect shifts in client physiology.

- **Paraverbal restraint**

° Abstinence from verbal fillers, nods, or affirmations during exposure, especially in the Silence Module.
° Reintroduction of prosodic reassurance only during the grounding phase to mark containment.

- **Neurophysiological monitoring**

° Ability to interpret HRV in real time.
° Discrimination between sympathetic arousal and dorsal collapse.
° Competence in initiating corrective grounding techniques if dissociation is detected.

- **Ethical and clinical vigilance**

° Capacity to terminate exposure upon detection of prodromal signs of psychosis, seizure aura, or acute suicidality.
° Basic life support certification and familiarity with rescue medication protocols.

## 4 Proposed methods

### 4.1 Standard session structure

All numeric values below are illustrative examples only and are intentionally non-normative.

**Phase 1** – **check-in (≈5 min, in a hypothetical protocol)**

- Assessment of distress on a 0–10 scale, recent sleep patterns, medication adherence, and acute psychosocial stressors.
- Narrative content is deliberately postponed to avoid early cortical activation.

**Phase 2** – **stimulus exposure (5**–**50 min)**

- **Silence module**: therapist and client remain seated without verbal or environmental stimuli (e.g., HVAC hum, digital devices). Visual contact is minimized. This may help train the ventral vagal complex to remain engaged in the absence of external cues. With progressive exposure duration, patients may exhibit increasing HRV, reflecting improved parasympathetic tone.
- **Sound Module**: therapist may administer sine wave tones beginning at 400 Hz/40 dB. Each could increase by ~40 Hz or 5 dB toward a ceiling of 700 Hz/65 dB. Therapist remains visible, signaling safety through calm posture and breath. The cyclical pattern of activation and recovery is intended to facilitate adaptive baroreflex sensitivity and autonomic feedback learning.
- **Isolation Module**: therapist exits following a synchronized breath cue. The room could be monitored via discreet video feed. This may foster transitions between mild sympathetic protest and self-generated soothing, thus potentially widening the individual's window of tolerance-i.e., the psychophysiological zone where affect is intense but manageable. The client may be equipped with a non-verbal emergency device to summon re-entry if necessary.

**Phase 3** – **grounding and re-entry (5**–**10 min)**

- Techniques may include diaphragmatic breathing at 0.1 Hz, proprioceptive tapping, slow ocular tracking, and gentle contralateral movement.
- Therapist language is restricted to sensorimotor anchoring (“Notice the floor beneath your feet,” etc.).

**Phase 4** – **micro-reflection (≤5 min)**

- Client offers a single-word or short-phrase descriptor of somatic state (e.g., “buzzing,” “dense,” “weightless”).
- No autobiographical linking is permitted at this stage to preserve interoceptive fidelity.

### 4.2 Module-specific technical details

- **Silence module**

° The acoustic environment must be carefully managed (e.g., studio-grade door seals, deactivated HVAC, devices on airplane mode).
° The therapist should minimize micro-noise (e.g., clothing movement, pen clicking).
° In a hypothetical implementation, progression may follow: “numbing fog” → “quiet tension” → “neutral presence.”

- **Sound module**

° Audio could be played as 24-bit WAV with 3-s fade-in and fade-out in future experimental implementations.
° The frequencies selected are within typical human alarm range (~500–800 Hz) but structured to maintain tonal continuity (Edworthy et al., 1991).
° Data could be collected via GSR, with reductions in latency to peak indicating progress.

- **Isolation module**

° Room setup may include a single chair, neutral decor, one analog clock; mirrors should be avoided.
° An emergency “stop” button could be included to activate an external light signal, prompting therapist re-entry within a predefined time frame (e.g., 10 s) in experimental settings.
° Client progression may shift from door pounding to pacing, seated agitation, then reflective stillness over sessions 1–2, 3–4, 5–7, and 8–10, respectively.

### 4.3 Clinical eligibility and exclusion criteria

Participants should meet the following inclusion criteria:

- DSM-5 diagnosis of BPD or ICD-11 Borderline Pattern.
- Demonstrated capacity to remain in a session for at least 20 min without elopement.
- Verbal and cognitive capacity to engage with distress scaling (IQ > 80).
- Psychopharmacological stability: SSRIs and SGAs permitted; benzodiazepine use restricted to ≤ 1 mg lorazepam equivalent within 24 h pre-session.

Exclusion Criteria are as follows:

- Diagnosed psychotic spectrum disorders (e.g., schizophrenia, schizoaffective).
- Active manic or hypomanic episode (Bipolar I).
- Uncontrolled epilepsy due to sound-triggered seizure risk in sound exposure sessions.
- Dissociative identity disorder with unintegrated switching.
- Severe PTSD with flashbacks to sensory deprivation or captivity.

### 4.4 Integration sessions (Weeks 11, 21, 31)

- **Narrative reconstruction**: patients verbalize their experience using metaphor or sensory-based language.
- **Symbolic processing**: therapist supports meaning-making of recurring themes or dreams.
- **Anchoring future practice**: patients assigned self-regulation tasks (e.g., stillness exercises, solo activities).

**Outcome metrics**: update self-report measures and interview-based coherence ratings (e.g., Adult Attachment Interview).

## 5 Hypothesized results

This threshold is illustrative for powering future pilot trials and does not represent a clinical expectation or efficacy claim.

### 5.1 Outcome markers

- **Session-level (micro outcomes)**

° HRV may return to baseline within 5 min of grounding.
° Self-reported distress ≤ 4/10 before session exit.

- **Program-level (macro outcomes – hypothetical targets for future trials)**

° ≥30% reduction in Zanarini Rating Scale score by Week 16 is proposed as a measurable outcome for future trials.
° ≥50% reduction in daily affective instability scores assessed via EMA (Trull et al., 2008).
° Post-isolation module gain of ≥1 point in AAI coherence.

When bodily arousal is no longer overwhelming, symbolic and narrative integration is hypothesized to begin to emerge more spontaneously. In this hypothetical model, repeated exposure to previously dysregulating conditions is expected to facilitate a gradual shift from undifferentiated threat and collapse toward more differentiated awareness of bodily sensations, sounds and interpersonal distance. Over time, this may be accompanied by an increased capacity to organize these experiences in images and words.

Furthermore, if autonomic hyperactivation declines, cortical areas dedicated to social cognition may regain function. Over time, this process is expected, within this hypothetical framework, to reduce misinterpretations of neutral expressions as hostile, foster greater perspective-taking and empathy, and support more effective conflict resolution.

## 6 Discussion

Neuroplastic change is thought to require prediction error: a discrepancy between the brain's expectations and actual outcomes (Friston, 2010; Bouton, 2004; Yau and McNally, 2023). When patients enter atmospheres historically coded as catastrophic (e.g., silence) and no harm ensues, maladaptive threat circuits—particularly in the basolateral amygdala—may begin to destabilize.

The structured dosing schedule of TTB—incremental increases such as +5 min (silence) or +40 Hz/+5 dB (sound)—is intended to optimize this window for learning without overwhelming the client. Within this framework, exposure is hypothesized to lead not only to habituation but also to memory reconsolidation: previously fear-associated memory traces may be rewritten into neutral or even positive representations (Schiller et al., 2010).

Physiologically, TTB hypothesizes that such a process might manifest as attenuated cortisol release across sessions; neurologically, as diminished limbic reactivity on neuroimaging; experientially, as reappraisals such as “tense but safe” rather than “void equals death” (Bouton, 2004; Yau and McNally, 2023). In this hypothetical model, the borderline feedback loop (internal chaos → interpersonal rupture → abandonment panic → impulsive action → guilt/shame → renewed chaos) may become disrupted. By teaching the body that early caregiver-mediated sensory-affective environments—prolonged silence, intense sound, and enforced isolation—are survivable:

- Silence no longer signals annihilation; thus, a partner's pause no longer triggers panic.
- Sound no longer equates to chaos, allowing arguments to remain discussions.
- Solitude becomes tolerable, reducing compulsive contact-seeking.

The result is hypothesized to be decreased volatility not via behavioral suppression, but through autonomic stabilization (Thayer and Lane, 2009; Beauchaine, 2015).

### 6.1 Affective processing without narrative contamination

Traumatic experiences in BPD often predate language or are stored in somatosensory formats inaccessible to verbal recall. Premature insistence on narrative coherence may provoke confabulation, reinforcing shame or cognitive distortion.

TTB delays autobiographical exploration until integration sessions, allowing right-hemisphere, sensory-encoded fragments to process somatically. During exposure, patients track micro-sensations—heat in the chest, vibrating limbs, visceral contractions—while the therapist anchors regulation through breath and presence.

This process is hypothesized to engage the insula and anterior cingulate cortex and to gradually build a more precise interoceptive map (Critchley and Garfinkel, 2017). Only once these foundational representations are integrated does figurative language emerge naturally—metaphors, symbolic imagery, or authentic memory shards—indicative of stable hemispheric transfer.

### 6.2 Controlled exposure vs. retraumatization

Concerns about retraumatization are valid in populations with affective instability. TTB embeds four systemic safeguards to reduce this risk:

1. **Predictable structure**: each session adheres to a consistent temporal framework, providing anticipatory safety.
2. **Graduated titration**: exposure intensity increases only when physiological stability is confirmed (e.g., HRV recovery, regulated gaze, coherent verbal output).
3. **Continuous co-regulation**: the therapist serves as a live autonomic anchor throughout all modules, including visible presence during sound and monitored proximity during isolation.
4. **Client agency**: a silent “stop” mechanism permits immediate withdrawal from any exposure, restoring control where trauma history often involved helplessness.

These features are intended to increase the likelihood that exposure experiences consolidate as mastery rather than retraumatization (Bouton, 2004; Yau and McNally, 2023).

### 6.3 Distress tolerance as structural reorganization

Whereas skills-based treatments (e.g., DBT) rely on top-down application of coping strategies, TTB aims to restructure bottom-up autonomic architecture (Thayer and Lane, 2009; Beauchaine, 2015).

Through repeated sessions, brainstem and limbic circuits may develop enhanced capacity to self-regulate. Patients may notice spontaneous somatic responses, e.g., slowed exhalation, relaxed musculature, before conscious regulation is engaged.

Like cardiovascular conditioning, once autonomic flexibility is trained it may deploy more automatically, freeing executive function for higher-level interpersonal and occupational functioning.

### 6.4 Clinical implications – hypothesis-driven considerations

TTB is, at present, an unvalidated theoretical construct. Its procedures derive from convergent domains, but no outcome studies have yet been conducted. Consequently, the following clinical implications must be interpreted as exploratory. Any therapeutic application of TTB should be undertaken within a clearly defined research framework, supported by data collection, ethical review, and robust safety protocols.

Preliminary case observations and theoretical coherence suggest that TTB may be appropriate for individuals who meet DSM-5 criteria for BPD, or who exhibit subclinical borderline traits marked by chronic autonomic dysregulation. Potential clinical profiles include:

- **Affective lability tied to sensory environments:** individuals who disintegrate in silence (“the void swallows me”), exhibit startle reactions to sharp sounds (“it feels like an attack”), or experience panic when alone for short periods.
- **Histories of neglect or disorganized caregiving:** those with backgrounds of adoption, institutional care, chronically depressed or substance-involved caregivers, or frequent changes in primary attachment figures.
- **Non-responsiveness to cognitive and dialectical treatments:** patients who demonstrate cognitive mastery of therapeutic skills but remain physiologically dysregulated in response to quiet, relational conflict, or solitude.
- **Complex trauma physiology**, including individuals with

- Complex PTSD
- Developmental trauma disorder
- Disorganized attachment profiles (e.g., on the Adult Attachment Interview)
- High levels of dissociation without coherent narrative recall

Inclusion decisions should prioritize physiological evidence of intolerance (e.g., reproducible HRV collapse during silence) over diagnostic labels alone. Proof-of-concept trials may benefit from stratifying participants based on baseline vagal tone to assess differential outcomes.

#### 6.4.1 Experimental integration pathways

Given its untested nature, TTB should be integrated within existing clinical frameworks to ensure comparative analysis and clinical safety:

1. **Pre-treatment priming:** delivering 10–20 TTB sessions before initiating DBT or schema therapy may optimize autonomic conditions for subsequent cognitive skill acquisition. *Suggested design:* randomized controlled sequences, and autonomic markers (e.g., HRV, startle reactivity) as mediating variables.
2. **Mid-treatment adjunct:** in long-term psychodynamic or relational therapies that reach somatic impasses, a short TTB module (e.g., six sessions) may restore affective integration. *Suggested method:* single-case experimental designs with multiple baselines.
3. **Intensive inpatient pilot:** ten daily sessions within a residential trauma unit, combined with 24-h biometric monitoring, may assist in acute stabilization. *Outcome metrics:* benzodiazepine usage, restraint incidents, nocturnal HRV recovery.
4. **Group-based exploratory labs (future direction):** small groups (e.g., four participants) simultaneously engage in silent or sound exposures, guided by dual clinicians. Group physiology (e.g., dyadic HRV synchrony) becomes a measurable endpoint.

All integration strategies must receive ethics board approval, define stopping criteria, and pre-register analysis plans to reduce experimental bias.

### 6.5 Contraindications and cautions

Due to uncertain risk parameters, clinical exclusion should err on the side of caution. Contraindicated populations include:

- **Current psychosis** (schizophrenia spectrum or manic episodes): sensory modulation may intensify hallucinations or grandiosity (Cloitre, 2009).
- **Dissociative identity disorder**: may provoke protector alter activation or fugue states.
- **Recent or active suicidality**: patients with attempts or clear intent should undergo stabilization before participation.
- **Substance intoxication or withdrawal**: autonomic flux interferes with stimulus titration and mimics trauma intrusions.
- **Neurological hypersensitivities**: e.g., temporal lobe epilepsy, Ménière's disease, or severe migraine with phonophobia.
- **Severe cardiac instability**: potential for arrhythmias during sympathetic activation phases.

Relative cautions include pregnancy (limit sound exposure intensity), uncontrolled hypertension (initiate at minimal exposure), and autism spectrum profiles with sound hypersensitivity (use bone conduction devices, adjust ramp-up schedule).

### 6.6 Potential future applications (research agenda)

The core mechanism of TTB—exposure to early caregiver-mediated sensory-affective environments—may have broader utility beyond BPD, including:

- **Complicated grief**: silence as an intolerable reminder of absence may become metabolizable through titrated exposure.
- **Occupational trauma**: firefighters or paramedics who dissociate in quiet post-shift settings may regain rest capacity.
- **Traumatized autistic individuals**: the sound module may improve tolerance to environmental auditory input.
- **Adolescents with attachment rupture**: the isolation module, paired with family therapy, may foster autonomous affect regulation.
- **Geriatric anxiety**: nocturnal silence linked to death anxiety may be gradually desensitized.

Pilot studies should employ mixed methods (e.g., physiological metrics + qualitative interviews) to capture subjective and objective change processes.

### 6.7 Ethical imperatives and safeguard strategies

Without efficacy data, clinical use of TTB must be regarded as experimental and research integrated. As such, enhanced informed consent must be gained. This includes verbal explanation, a plain-language information sheet, and a brief video overview. Furthermore, patients' ongoing consent must be confirmed before each exposure, and it should be clear that dissent halts the process without any repercussions.

Every session should end with a 10-min recovery period. This could include a warm beverage, weighted blanket, and/or grounding aids. All unexpected responses, e.g., dizziness, flashbacks, and dissociation, should be recorded for pooled safety analysis.

Untrained clinicians may attempt unsafe DIY adaptations. Therefore, trademarked certification processes, fidelity checklists, and public practitioner registries should be used for recruitment. Additionally, sustained silent co-regulation is taxing even with adequate training. Therefore, rotation scheduling, HRV biofeedback, and mindfulness practices for therapists should be in place to avoid clinician fatigue. Furthermore, monthly peer review of video-recorded sessions can support fidelity and mitigate therapist overreach.

At this stage, TTB must be regarded not as a validated clinical method, but as a testable hypothesis. Both successful and null results must be submitted to peer-reviewed outlets to counteract publication bias.

### 6.8 Limitations and future research

TTB occupies the developmental stage characteristic of all emerging clinical models: rich in conceptual scaffolding, yet empirically unsubstantiated. The following section critically evaluates key limitations and outlines a multidimensional research agenda aimed at rigorously testing whether this neuroaffective framework can transition from theoretical innovation to evidence-based intervention. Limitations and proposals are organized across eight domains: methodological, therapist-related, client-related, ethical, cross-diagnostic, cultural-contextual, technological, and logistical. Each concludes with actionable research recommendations.

#### 6.8.1 Absence of empirical data

##### 6.8.1.1 Current gap

To date, no randomized controlled trials, controlled case series, or formal single-case experimental designs have evaluated TTB. While anecdotal accounts and phenomenological field notes provide clinical insight, they cannot eliminate confounding variables such as placebo response, expectancy bias, or therapist charisma. Neurophysiological claims (e.g., vagal tone modulation, limbic reconsolidation) remain speculative in the absence of direct measurement, despite converging evidence for related mechanisms in research on heart rate variability and prediction-error-based learning (Thayer and Lane, 2009; Bouton, 2004; Yau and McNally, 2023).

In addition, TTB is explicitly grounded in broader theoretical frameworks, such as polyvagal theory and the somatic marker hypothesis, whose empirical status is themselves debated; both have attracted substantial conceptual and methodological criticism regarding the strength and specificity of the available evidence (Dunn et al., 2006; Grossman and Taylor, 2007; Liem and Neuhuber, 2021; Linquist and Bartol, 2013). Accordingly, TTB is proposed as a provisional integrative model that draws on these frameworks while explicitly acknowledging that their empirical foundations continue to be refined and debated.

##### 6.8.1.2 Research tasks

1. Pilot feasibility studies with *N* = 8–12 participants to assess completion rates, safety parameters, and preliminary effect sizes across autonomic and self-report metrics.
2. Multiple-baseline single-case designs, staggering intervention onset to differentiate treatment effects from natural maturation.
3. Observational cohort comparisons: TTB plus treatment as usual vs. treatment as usual alone.

Key outcome domains:

- Behavioral (e.g., frequency of self-injury, crisis service utilization).
- Psychophysiological (e.g., resting HRV, acoustic startle response).
- Experiential (e.g., ecological momentary assessment of affective states).

#### 6.8.2 Therapist variability

##### 6.8.2.1 Current gap

TTB demands high-level competencies—sustained non-verbal attunement, silent co-regulation, detection of subtle autonomic shifts—which are unevenly distributed among practitioners. Early positive outcomes may reflect individual therapist strengths rather than intervention fidelity. Without standardized protocols and monitoring, reproducibility remains uncertain.

##### 6.8.2.2 Research tasks

1. Delphi studies to define core therapist competencies (e.g., HRV interpretation, somatic containment skills).
2. Development and validation of fidelity checklists assessing session parameters (e.g., exposure dose, regulatory stance, grounding techniques), rated by independent observers.
3. Therapist effect audits across multiple sites to assess inter-practitioner variance.
4. Monitoring of therapist wellbeing: weekly administration of compassion fatigue scales, countertransference logs, and burnout indices.

#### 6.8.3 Risk of affect overexposure

##### 6.8.3.1 Current gap

Although gradual dosing is embedded in the protocol, some individuals may dissociate, experience autonomic shutdown, or develop paradoxical sensitization (e.g., heightened reactivity to previously neutral stimuli). The absence of early verbal scaffolding may intensify distress in patients with high cognitive needs, potentially reinforcing helplessness.

##### 6.8.3.2 Research tasks

1. Dose–response calibration studies to identify physiological overload thresholds (e.g., >30% HRV drop, GSR spikes >2 μS).
2. Real-time safety algorithms using machine learning to detect biometric collapse and prompt therapist intervention.
3. Post-session sensitization tracking via structured follow-up surveys (24–48 h post-exposure).
4. Controlled studies on adjunct verbal anchoring: random assignment to silent-only vs. verbal-titrated conditions to assess mitigation of overexposure effects.

#### 6.8.4 Ethical tension in sensory manipulation

##### 6.8.4.1 Current gap

The sensory elements of TTB may inadvertently echo coercive environments (e.g., solitary confinement, verbal abuse). Misapplication of the protocol, in the absence of oversight, may retraumatize rather than heal.

##### 6.8.4.2 Research tasks

1. Institutional Review Board-approved templates detailing exposure content, opt-out mechanisms, and adverse event response protocols.
2. Inclusion of client advisory panels with lived experience to co-develop stimulus content, safety language, and distress signals.
3. Informed consent comprehension assessments ensuring patients fully understand the experimental nature of TTB, anticipated discomforts, and right to withdraw.
4. Session-by-session ethical acceptability ratings to track client perception over time and identify when protocol modifications are necessary.

#### 6.8.5 Generalization across diagnostic spectra

##### 6.8.5.1 Current gap

Though TTB was conceived for individuals with borderline traits, its applicability to other disorders is undetermined. Patients with avoidant, narcissistic, obsessive-compulsive traits or mood and neurodevelopmental conditions may exhibit divergent responses to sensory-based interventions, due to differing affective styles or autonomic profiles.

##### 6.8.5.2 Research tasks

1. Pilot studies with small cohorts representing diverse diagnostic groups (e.g., complex PTSD, avoidant personality disorder, autism with trauma overlay).
2. Moderator analyses exploring whether variables such as attachment style, sensory sensitivity, or alexithymia predict treatment response.
3. Transdiagnostic module testing: implementation of mini-exposures (e.g., silence-only or sound-only) across diagnostic groups to isolate mechanisms of action.

#### 6.8.6 Cultural and contextual limitations

##### 6.8.6.1 Current gap

Interpretations of silence and isolation vary across cultural settings. What is perceived as restorative in one culture may evoke neglect or punishment in another. Additionally, environmental baselines—such as urban noise or multigenerational living—may alter stimulus tolerability.

##### 6.8.6.2 Research tasks

1. Ethnographic studies to explore culturally specific meanings attributed to silence, sound, and solitude.
2. Cross-cultural adaptation guidelines for modifying stimulus parameters (e.g., intensity, duration) and interpretive framing.
3. Multi-site international research comparing outcomes in collectivist vs. individualist societies, with attention to cultural idioms of distress.
4. Socioeconomic moderation analyses: testing whether environmental variables (e.g., housing density, digital media exposure) influence stimulus tolerance.

#### 6.8.7 Technological and measurement limitations

##### 6.8.7.1 Current gap

TTB requires access to controlled acoustic environments, high-resolution HRV and GSR capture, and real-time visual monitoring, all of which may exceed the capabilities of community-based clinics. Wearable sensors remain prone to motion artifacts, and HRV algorithms may not generalize across skin tones or body types.

##### 6.8.7.2 Research tasks

1. Evaluation of low-resource adaptations (e.g., mobile sound booths, smartphone-based HRV monitors, bone conduction audio).
2. Instrumentation validation studies comparing commercial devices with gold-standard equipment (e.g., ECG) during exposure tasks.
3. Data security audits to simulate breaches and improve biometric data encryption; concurrent client surveys on privacy acceptability.
4. Development of AI-assisted titration tools: open-source platforms that dynamically adjust exposure intensity based on real-time biometric input.

#### 6.8.8 Logistical and Funding Challenges

##### 6.8.8.1 Current gap

TTB's implementation entails considerable costs: soundproof rooms, specialized training, biometric sensors, and prolonged session durations. These demands often exceed the infrastructure and budgets of outpatient settings. Furthermore, insurance coverage for unvalidated protocols is unlikely.

##### 6.8.8.2 Research tasks

1. Cost-effectiveness simulations modeling long-term savings (e.g., reduced hospitalization, emergency interventions) to justify funding.
2. Staged grant acquisition: beginning with pilot funding from trauma-focused foundations, followed by larger multi-site applications upon demonstration of feasibility.
3. Public-private partnerships with technology firms producing wearable and VR tools relevant to sensory modulation.
4. Formation of interinstitutional training hubs to pool resources and centralize expertise, avoiding duplication of effort across clinics.

#### 6.8.9 Recommendations for early research

**Phase I (0–2 years)**

- Conduct ≤ 15 multiple-baseline single-case studies with rigorous autonomic tracking.
- Publish all outcomes (positive or null) to mitigate publication bias.
- Finalize a fidelity checklist and develop standardized therapist training with competency-based assessments.

**Phase II (2–5 years)**

- Initiate a two-site RCT (TTB + standard care vs. standard care) powered for autonomic markers (e.g., HRV, startle reactivity).
- Pilot a VR-assisted TTB prototype to enhance dosing precision and examine user acceptability.

**Phase III (5–10 years)**

- Launch a pragmatic multi-site RCT (N ≥ 120), assessing symptom remission (e.g., Zanarini Rating Scale), functional outcomes (e.g., employment, social integration), and 12-month sustainability.
- Perform stratified analyses by attachment style, trauma burden, and cultural variables to refine inclusion criteria.

**Long-term goals**

- Establish an open-access biometric and outcomes repository to accelerate meta-analyses and cross-site synthesis.
- Formulate clinical policy recommendations governing the ethical implementation of sensory exposure therapies.

## 7 Conclusion

TTB stands at the threshold of scientific possibility—conceptually provocative, yet presently lacking the empirical foundation required for broad clinical adoption. The articulation of its limitations should not be seen as a dismissal but rather as an invitation for rigorous exploration and collaborative scrutiny. Scientific progress, especially in clinical innovation, demands not only the courage to propose new frameworks but also the humility to subject them to systematic challenge, refinement, and empirical validation.

The true value of TTB will ultimately be determined not by the willingness of the scientific community to rigorously interrogate its premises, test its mechanisms, and clarify its scope of application. This process must include carefully designed pilot studies, randomized controlled trials, and multimodal assessments that extend beyond subjective reports to encompass physiological, behavioral, and experiential data. Only through such disciplined empirical efforts can we discern whether the model's proposed neuroaffective interventions offer substantial, reproducible benefits over existing treatments, or whether its effects are limited to specific clinical profiles or contexts.

Cultural humility is equally vital in this journey. The affective meanings of silence, sound, and solitude vary widely across cultural backgrounds, developmental histories, and individual experiences. As such, cross-cultural research, participatory design with lived-experience consultants, and the adaptation of protocols to diverse settings are essential to avoid ethnocentric bias and to ensure that TTB's methods are safe, relevant, and respectful for all populations. This sensitivity may not only enhance the ethical implementation of TTB but also foster a more nuanced understanding of how primary affective experiences shape psychopathology across the human spectrum.

Ethical vigilance must also be at the forefront of any experimental application. Researchers and clinicians must establish transparent consent procedures, robust safety monitoring, and clear stopping rules to protect participants—particularly those who may be vulnerable to overexposure or retraumatization. Regular supervision, peer review, and open sharing of both positive and negative outcomes will promote transparency and counteract publication bias.

In sum, the next phase of TTB's development rests upon the collective commitment to methodological rigor, ethical responsibility, and clinical humility. If, through disciplined investigation, the protocol proves capable of recalibrating the autonomic imprints of trauma—transforming historically intolerable states into survivable, even restorative experiences—it may indeed provide a foundational advance in the treatment of borderline personality disorder. Until such evidence emerges, however, TTB should be regarded as a promising hypothesis: a potential catalyst for innovation, best pursued in partnership with critical inquiry and empirical scrutiny. Whether calibrated silence, modulated sound, and structured solitude can rewrite the deepest layers of human affective experience remains an open question—one worthy of sustained, rigorous pursuit.

## Data Availability

The original contributions presented in the study are included in the article/supplementary material, further inquiries can be directed to the corresponding author.

## References

[B1] Alberini C. M. LeDoux J. E. (2013). Memory reconsolidation. Curr. Biol. 23, R746–R750. doi: 10.1016/j.cub.06.046 24028957

[B2] Baptista A. Cohen D. Jacquet P. (2021). The cognitive, ecological, and developmental origins of self-disturbance in borderline personality disorder. Front. Psychiatry 12:707091. doi: 10.3389/fpsyt.2021.707091 34658950 PMC8514658

[B3] Barnicot K. Katsakou C. Marougka S. Priebe S. (2011). Treatment completion in psychotherapy for borderline personality disorder: a systematic review and meta-analysis. Acta Psychiatr. Scan. 123, 327–338. doi: 10.1111/j.1600-0447.2010.01652.x 21166785

[B4] Bateman A. Fonagy P. (2010). Mentalization-based treatment for borderline personality disorder. World Psychiatry 9, 11–15. doi: 10.1002/j.2051-5545.2010.tb00255.x 20148147 PMC2816926

[B5] Beauchaine T. P. (2015). Respiratory sinus arrhythmia: a transdiagnostic biomarker of emotion dysregulation and psychopathology. Curr. Opin. Psychol. 3, 43–47. doi: 10.1016/j.copsyc.01.017 25866835 PMC4389219

[B6] Bohus M. Kleindienst N. Limberger M. F. Stieglitz R. D. Domsalla M. Chapman A. L. . (2009). The short version of the Borderline Symptom List (BSL 23): development and initial data on psychometric properties. Psychopathology 42, 32–39. doi: 10.1159/000173701 19023232

[B7] Bouton M. E. (2004). Context and behavioral processes in extinction. Learn. Memory 11, 485–494. doi: 10.1101/lm.78804 15466298

[B8] Bungert M. Liebke L. Thome J. Haeussler K. Bohus M. Lis S. . (2015). Pain processing after social exclusion and its relation to rejection sensitivity in borderline personality disorder. PLOS ONE 10:e0133693. doi: 10.1371/journal.pone.0133693 26241850 PMC4524681

[B9] Carozza S. Leong V. (2021). The role of affectionate caregiver touch in early neurodevelopment and parent-infant interactional synchrony. Front. Neurosci. 14:613378. doi: 10.3389/fnins.2020.613378 33584178 PMC7873991

[B10] Casellas Pujol E. Soler J. Schmidt C. Soria Madrid A. Elices M. Pascual J. C. . (2024). Contextual compassion training for borderline personality disorder with long lasting symptoms: a randomized clinical trial. J. Context. Behav. Sci. 34:100846. doi: 10.1016/j.jcbs.2024.100846

[B11] Chaudhury S. R. Galfalvy H. Biggs E. Choo T. H. Mann J. J. Stanley B. . (2017). Affect in response to stressors and coping strategies: an ecological momentary assessment study of borderline personality disorder. Borderline Personality Disord. Emotion Dysregulation 4:8. doi: 10.1186/s40479-017-0059-3 28533905 PMC5438851

[B12] Cloitre M. (2009). Effective psychotherapies for posttraumatic stress disorder: a review and critique. CNS Spectr. 14, 32–43. 19169192

[B13] Craske M. G. Kircanski K. Zelikowsky M. Mystkowski J. Chowdhury N. Baker A. . (2008). Optimizing inhibitory learning during exposure therapy. Behav. Res. Ther. 46, 5–27. doi: 10.1016/j.brat.10.003 18005936

[B14] Craske M. G. Treanor M. Conway C. C. Zbozinek T. Vervliet B. (2014). Maximizing exposure therapy: an inhibitory learning approach. Behav. Res. Ther. 58, 10–23. doi: 10.1016/j.brat.04.006 24864005 PMC4114726

[B15] Critchley H. D. Garfinkel S. N. (2017). Interoception and emotion. Curr. Opin. Psychol. 17, 7–14. doi: 10.1016/j.copsyc.04.020 28950976

[B16] Cruciani G. Boccia M. Lingiardi V. Giovanardi G. Zingaretti P. Spitoni G. F. . (2021). An exploratory study on resting-state functional connectivity in individuals with disorganized attachment: evidence for key regions in amygdala and hippocampus. Brain Sci. 11:1539. doi: 10.3390/brainsci11111539 34827538 PMC8615787

[B17] Cruciani G. Zingaretti P. De Filippis S. Zanini L. Iosa M. Lingiardi V. . (2025). In search for affect in painful touch. J. Affect. Disord. 380, 375–383. doi: 10.1016/j.jad.03.098 40147613

[B18] Cruciani G. Zingaretti P. Lingiardi V. De Filippis S. Haggard P. Spitoni G. F. . (2023). The perception of pain, discriminative touch and affective touch in patients suffering from borderline personality disorder. J. Affect. Disord. 341, 185–193. doi: 10.1016/j.jad.08.126 37657618

[B19] Damasio A. (1999). The Feeling of what Happens: Body and Emotion in the Making of Consciousness. New York, NY: Harcourt Brace.

[B20] Dunn B. D. Dalgleish T. Lawrence A. D. (2006). The somatic marker hypothesis: a critical evaluation. Neurosci. Biobehav. Rev. 30, 239–271. doi: 10.1016/j.neubiorev.07.001 16197997

[B21] Edworthy J. Loxley S. Dennis I. (1991). Improving auditory warning design: relationship between warning sound parameters and perceived urgency. Human Factors 33, 205–231. doi: 10.1177/001872089103300206 1860703

[B22] Farina B. Speranza A. M. Imperatori C. Quintiliani M. I. Della Marca G. (2015). Change in heart rate variability after the Adult Attachment Interview in dissociative patients. J. Trauma Dissociation 16, 170–180. doi: 10.1080/15299732.2014.975309 25492425

[B23] Feldman R. (2017). The neurobiology of human attachments. Trends Cogn. Sci. 21, 80–99. doi: 10.1016/j.tics.11.007 28041836

[B24] Feldman R. (2020). What is resilience: an affiliative neuroscience approach. World Psychiatry 19, 132–150. doi: 10.1002/wps.20729 32394561 PMC7215067

[B25] Fonagy P. Gergely G. Jurist E. L. Target M. (2018). Affect Regulation, Mentalization and the Development of the Self . London: Routledge. doi: 10.4324/9780429471643

[B26] Fonagy P. Luyten P. Allison E. (2015). Epistemic petrification and the restoration of epistemic trust: a new conceptualization of borderline personality disorder and its psychosocial treatment. J. Pers. Disord. 29, 575–609. doi: 10.1521/pedi.2015.29.5.575 26393477

[B27] Fonagy P. Luyten P. Allison E. Campbell C. (2017). What we have changed our minds about: part 2. Borderline personality disorder, epistemic trust and the developmental significance of social communication. Borderline Pers. Disord. Emotion Dysregulation 4:9. doi: 10.1186/s40479-017-0062-8 28405338 PMC5387344

[B28] Francis D. D. Diorio J. Liu D. Meaney M. J. (1999). Nongenomic transmission across generations of maternal behaviour and stress responses in the rat. Science 286, 1155–1158. doi: 10.1126/science.286.5442.1155 10550053

[B29] Friston K. (2010). The free-energy principle: a unified brain theory? Nat. Rev. Neurosci. 11, 127–138. doi: 10.1038/nrn2787 20068583

[B30] Gilbert P. (2014). The origins and nature of compassion focused therapy. Brit. J. Clin. Psychol. 53, 6–41. doi: 10.1111/bjc.12043 24588760

[B31] Grossman P. Taylor E. W. (2007). Toward understanding respiratory sinus arrhythmia: relations to cardiac vagal tone, evolution and biobehavioral functions. Biol. Psychol. 74, 263–285. doi: 10.1016/j.biopsycho.11.014 17081672

[B32] Hazlett E. A. Zhang J. New A. S. Zelmanova Y. Goldstein K. E. Haznedar M. M. . (2012). Potentiated amygdala response to repeated emotional pictures in borderline personality disorder. Biol. Psychiatry 72, 448–456. doi: 10.1016/j.biopsych.03.027 22560044 PMC3415575

[B33] Kamphausen S. Schröder P. Maier S. Bader K. Feige B. Kaller C. P. . (2013). Medial prefrontal dysfunction and prolonged amygdala response during instructed fear processing in borderline personality disorder. World J. Biol. Psychiatry 14, 307–318. doi: 10.3109/1562012.665174 22404662

[B34] Kivity Y. Levy K. N. Kolly S. Kramer U. (2020). The therapeutic alliance over 10 sessions of therapy for borderline personality disorder: agreement and congruence analysis and relation to outcome. J. Pers. Disord. 34, 1–21. doi: 10.1521/pedi_2019_33_376 30689513

[B35] Koenig J. Kemp A. H. Feeling N. R. Thayer J. F. Kaess M. (2016). Resting state vagal tone in borderline personality disorder: a meta-analysis. Prog. Neuro Psychopharmacol. Biol. Psychiatry 64, 18–26. doi: 10.1016/j.pnpbp.07.002 26169575

[B36] Koenigsberg H. W. Denny B. T. Fan J. Liu X. Guerreri S. Mayson S. J. . (2014). The neural correlates of anomalous habituation to negative emotional pictures in borderline and avoidant personality disorder patients. Am. J. Psychiatry 171, 82–90. doi: 10.1176/appi.ajp.2013.13070852 24275960 PMC3947284

[B37] Koole S. L. Tschacher W. (2016). Synchrony in psychotherapy: a review and an integrative framework for the therapeutic alliance. Front. Psychol. 7:862. doi: 10.3389/fpsyg.2016.00862 27378968 PMC4907088

[B38] Krause Utz A. Walther J. C. Lis S. Schmahl C. Bohus M. (2019). Heart rate variability during a cognitive reappraisal task in female patients with borderline personality disorder: the role of comorbid posttraumatic stress disorder and dissociation. Psychol. Med. 49, 1810–1821. doi: 10.1017/S0033291718002489 30198447 PMC6650777

[B39] Lane R. D. Ryan L. Nadel L. Greenberg L. (2015). Memory reconsolidation, emotional arousal, and the process of change in psychotherapy: new insights from brain science. Behav Brain Sci. 38:e1. doi: 10.1017/S0140525X14000041 24827452

[B40] Lans L. L. Huijbregts K. M. L. Westerhof G. J. Haeyen S. W. Derks Y. P. M. J. Noordzij M. L. . (2025). How to integrate physiological data from wearables in treatment of personality disorders: a narrative review. Front. Psychiatry 16:1591871. doi: 10.3389/fpsyt.2025.1591871 40606826 PMC12218767

[B41] LeDoux J. E. (2002). Synaptic Self: How our Brains Become Who We Are. New York, NY: Viking.

[B42] Lehrer P. M. Kaur K. Sharma A. Shah K. Huseby R. Bhavsar J. . (2020). Heart rate variability biofeedback improves emotional and physical health and performance: a systematic review and meta-analysis. Appl. Psychophysiol. Biofeedback 45, 109–129. doi: 10.1007/s10484-020-09466-z 32385728

[B43] Leichsenring F. Fonagy P. Heim N. Kernberg O. F. Leweke F. Luyten P. . (2024). Borderline personality disorder: a comprehensive review of diagnosis and clinical presentation, etiology, treatment and current controversies. World Psychiatry 23, 4–25. doi: 10.1002/wps.21156 38214629 PMC10786009

[B44] Levy K. N. Meehan K. B. Kelly K. M. Reynoso J. S. Weber M. Clarkin J. F. . (2006). Change in attachment patterns and reflective function in a randomized control trial of transference focused psychotherapy for borderline personality disorder. J. Consulting Clin. Psychol. 74, 1027–1040. doi: 10.1037/0022-006X.74.6.1027 17154733

[B45] Liem T. Neuhuber W. (2021). Kritik an der Polyvagal theorie [Critique of the polyvagal theory]. Deutsche Zeitschrift für Osteopathie 19, 34–37. doi: 10.1055/a-1345-6051

[B46] Linehan M. M. (1993). Cognitive-Behavioral Treatment of Borderline Personality Disorder. New York, NY: Guilford Press.

[B47] Linquist S. Bartol J. (2013). Two myths about somatic markers. Brit. J. Philos. Sci. 64, 455–484. doi: 10.1093/bjps/axs020

[B48] Löffler A. Kleindienst N. Neukel C. Bekrater-Bodmann R. Flor H. (2022). Pleasant touch perception in borderline personality disorder and its relationship with disturbed body representation. Borderline Pers. Disorder Emotion Dysregulation 9:3. doi: 10.1186/s40479-021-00176-4 35101119 PMC8805331

[B49] Lyons-Ruth K. Pechtel P. Yoon S. A. Anderson C. M. Teicher M. H. (2016). Disorganized attachment in infancy predicts greater amygdala volume in adulthood. Behav. Brain Res. 308, 83–93. doi: 10.1016/j.bbr.03.050 27060720 PMC5017306

[B50] McLaughlin K. A. Sheridan M. A. Lambert H. K. (2014). Childhood adversity and neural development: deprivation and threat as distinct dimensions of early experience. Neurosci. Biobehav. Rev. 47, 578–591. doi: 10.1016/j.neubiorev.10.012 25454359 PMC4308474

[B51] Milad M. R. Quirk G. J. (2012). Fear extinction as a model for translational neuroscience: ten years of progress. Ann. Rev. Psychol. 63, 129–151. doi: 10.1146/annurev.psych.121208.131631 22129456 PMC4942586

[B52] Miller C. E. Townsend M. L. Grenyer B. F. S. (2021). Understanding chronic feelings of emptiness in borderline personality disorder: a qualitative study. Borderline Pers. Disorder Emotion Dysregulation 8:24. doi: 10.1186/s40479-021-00164-8 34365966 PMC8351135

[B53] Ogden P. Minton K. Pain C. (2006a). A sensorimotor approach to the treatment of trauma and dissociation. Psychiatric Clin. North Am. 29, 263–279. doi: 10.1016/j.psc.10.012 16530597

[B54] Ogden P. Minton K. Pain C. (2006b). Trauma and the Body: A Sensorimotor Approach to Psychotherapy. New York, NY: W. W. Norton and Company.

[B55] Payne P. Levine P. A. Crane-Godreau M. A. (2015). Somatic experiencing: using interoception and proprioception as core elements of trauma therapy. Front. Psychol. 6:93. doi: 10.3389/fpsyg.2015.00093 25699005 PMC4316402

[B56] Porges S. W. (2011). The Polyvagal Theory: Neurophysiological Foundations of Emotions, Attachment, Communication, Self-Regulation. New York, NY: W. W. Norton and Company.

[B57] Porges S. W. (2022). Polyvagal theory: a science of safety. Front. Integr. Neurosci. 16:871227. doi: 10.3389/fnint.2022.871227 35645742 PMC9131189

[B58] Porter C. Palmier-Claus J. Branitsky A. Mansell W. Warwick H. Varese F. . (2020). Childhood adversity and borderline personality disorder: a meta-analysis. Acta Psychiatr. Scan. 141, 6–20. doi: 10.1111/acps.13118 31630389

[B59] Pourmand V. Froidevaux N. M. Williams D. P. Yim I. S. Campos B. (2023). Attachment insecurity, heart rate variability, and perceived social support in a diverse sample of young adults. Front. Psychol. 14:1208924. doi: 10.3389/fpsyg.2023.1208924 38023002 PMC10667911

[B60] Preti E. Richetin J. Poggi A. Fertuck E. A. (2023). A model of trust processes in borderline personality disorder: a systematic review. Curr. Psychiatry Rep. 25, 555–567. doi: 10.1007/s11920-023-01468-y 37889465 PMC10654201

[B61] Ramseyer F. Tschacher W. (2011). Nonverbal synchrony in psychotherapy: coordinated body movement reflects relationship quality and outcome. J. Consult. Clin. Psychol. 79, 284–295. doi: 10.1037/a0023419 21639608

[B62] Schiller D. Monfils M.-H. Raio C. M. Johnson D. C. LeDoux J. E. Phelps E. A. (2010). Preventing the return of fear in humans using reconsolidation update mechanisms. Nature 463, 49–53. doi: 10.1038/nature08637 20010606 PMC3640262

[B63] Schore A. N. (2003). Affect Dysregulation and Disorders of the Self . New York, NY: W. W. Norton and Company.

[B64] Schore A. N. (2012). The Science of the Art of Psychotherapy. New York, NY: W. W. Norton and Company.

[B65] Spitoni G. F. Zingaretti P. Giovanardi G. Antonucci G. Galati G. Lingiardi V. . (2020). Disorganized attachment pattern affects the perception of affective touch. Sci. Rep. 10:9658. doi: 10.1038/s41598-020-66606-5 32541672 PMC7295781

[B66] Strauss T. Rottstädt F. Sailer U. Schellong J. Hamilton J. P. Raue C. . (2019). Touch aversion in patients with interpersonal traumatization. Depression Anxiety 36, 635–646. doi: 10.1002/da.22914 31209965

[B67] Suomi S. J. (1997). Early determinants of behavior: evidence from primate studies. Br. Med. Bull. 53, 170–184. doi: 10.1093/oxfordjournals.bmb.a011598 9158292

[B68] Teicher M. H. Samson J. A. (2016). Annual research review: enduring neurobiological effects of childhood abuse and neglect. J. Child Psychol. Psychiatry 57, 241–266. doi: 10.1111/jcpp.12507 26831814 PMC4760853

[B69] Thayer J. F. Lane R. D. (2009). Claude Bernard and the heart-brain connection: further elaboration of a model of neurovisceral integration. Neurosci. Biobehav. Rev. 33, 81–88. doi: 10.1016/j.neubiorev.08.004 18771686

[B70] Tomoda A. Sheu Y. S. Rabi K. Suzuki H. Navalta C. P. Polcari A. . (2011). Exposure to parental verbal abuse is associated with increased gray matter volume in superior temporal gyrus. NeuroImage 54, S280–S286. doi: 10.1016/j.neuroimage.05.027 20483374 PMC2950228

[B71] Trull T. J. Solhan M. B. Tragesser S. L. Jahng S. Wood P. K. Piasecki T. M. . (2008). Affective instability: measuring a core feature of borderline personality disorder with ecological momentary assessment. J. Abnormal Psychol. 117, 647–661. doi: 10.1037/a0012532 18729616

[B72] Tschacher W. Meier D. (2020). Physiological synchrony in psychotherapy sessions. Psychother. Res. 30, 558–573. doi: 10.1080/10503307.2019.1612114 31060474

[B73] van der Kolk B. A. (2014). The Body Keeps the Score: Brain, Mind, and Body in the Healing of Trauma. New York, NY: Viking.

[B74] van Harmelen A.-L. van Tol M.-J. van der Wee N. J. A. Veltman D. J. Aleman A. Spinhoven P. . (2010). Reduced medial prefrontal cortex volume in adults reporting childhood emotional maltreatment. Biol. Psychiatry 68, 832–838. doi: 10.1016/j.biopsych.06.011 20692648

[B75] van Hoof M. J. Riem M. M. E. Garrett A. S. van der Wee N. J. A. van IJzendoorn M. H. Vermeiren R. R. M. (2019). Unresolved-disorganized attachment adjusted for a general psychopathology factor associated with atypical amygdala resting-state functional connectivity. Eur. J. Psychotraumatol. 10:1583525. doi: 10.1080/20008198.2019.1583525 30891161 PMC6419678

[B76] Widiger T. A. Trull T. J. (2007). Plate tectonics in the classification of personality disorder: shifting to a dimensional model. Am. Psychol. 62, 71–83. doi: 10.1037/0003-066X.62.2.71 17324033

[B77] Yau J. O.-Y. McNally G. P. (2023). The Rescorla-Wagner model, prediction error, and fear learning. Neurobiol. Learn. Memory 203:107799. doi: 10.1016/j.nlm.2023.107799 37442411

[B78] Young J. E. Klosko J. S. Weishaar M. E. (2003). Schema Therapy: A Practitioner's Guide. New York, NY: Guilford Press.

[B79] Zanarini M. C. Frankenburg F. R. Reich D. B. Fitzmaurice G. (2010). Attainment of remission and recovery in borderline personality disorder: a 10-year follow-up study. Am. J. Psychiatry 167, 663–667. doi: 10.1176/appi.ajp.2009.09081130 20395399 PMC3203735

[B80] Zanarini M. C. Vujanovic A. A. Parachini E. A. Boulanger J. L. Frankenburg F. R. Hennen J. . (2003). Zanarini rating scale for borderline personality disorder (ZAN-BPD): a continuous measure of DSM-IV borderline psychopathology. J. Pers. Disorders 17, 233–242. doi: 10.1521/pedi.17.3.233.22147 12839102

[B81] Zingaretti P. Giovanardi G. Cruciani G. Lingiardi V. Ottaviani C. Spitoni G. F. . (2020). Heart rate variability in response to the recall of attachment memories. Attachment Human Dev. 22, 643–652. doi: 10.1080/1462019.1680712 31646928

